# Mechanical ventilation variables associated with high pulmonary artery pressures in ARDS patients: a post hoc analysis

**DOI:** 10.1186/s13054-022-04282-9

**Published:** 2022-12-21

**Authors:** Joseph R. Riddell, Benjamin J. Jones, Bruno M. Fernandes, Daniel J. Law, Jackie A. Cooper, Matt P. Wise

**Affiliations:** 1 Critical Care Department, University Hospital of Wales, Heath Park, Cardiff, CF14 4XW UK; 2 University College London, Gower Street, London, WC1E 6BT UK

## Abstract

**Background:**

The relationship between indices of mechanical ventilation and pulmonary artery pressures remains ill-defined in ARDS. As our understanding of mechanical ventilation has progressed, there is now a greater appreciation of the impact of high driving pressures and mechanical power in perpetuating lung injury. However, the relationship between the newer derived indices of mechanical ventilation and pulmonary artery pressure is unclear. We performed a post hoc analysis of the Fluid and Catheters Treatment Trial (FACTT) trial to investigate the associations between mechanical ventilation indices in ARDS patients and the prevalence of pulmonary hypertension. This may help elucidate future clinical targets for more, right ventricular protective, mechanical ventilation strategies.

**Methods:**

We performed a post hoc analysis of the FACTT database to identify ARDS patients who had a pulmonary artery catheter (PAC) inserted and pulmonary artery pressure readings recorded. We excluded any patient with a PAC inserted who was spontaneously breathing, as driving pressure and mechanical power are not validated in this cohort. Three independent analyses were performed: a univariate analysis, to assess for associations between mPAP and mechanical ventilation parameters using Pearson correlation coefficients, a multivariate analysis, to assess for independent associations with mPAP using a multiple regression model according to Akaike’s information criteria and finally an analysis for nonlinearity, using the best-fitting model according to the Bayesian information criterion (BIC) from linear, quadratic, fractional polynomial and restricted cubic spline models.

**Results:**

All the ventilation parameters demonstrated a significant correlation with mPAP, except tidal volume (once adjusted for respiratory rate) in the univariate analysis. The multivariate analysis demonstrated that the blood pH level, P/F ratio, PaCO_2_ level, mean airway pressure and the mechanical power indexed to compliance were independently associated with mPAP. In the final nonlinear analysis, associations did not differ from linearity except for 4 variables for which the fractional polynomial was the best-fitting model. These were mechanical power (*p* = 0.01 compared to the linear model), respiratory rate (*p* = 0.04), peak pressure (*p* = 0.03) and mean airway pressure (*p* = 0.01). Two nonlinear variables associated with mPAP were assessed in more detail, respiratory rate and mechanical power. Inflexion points at a respiratory rate of 16.8 cycles per minute and a mechanical power of 8.8 J/min were demonstrated.

**Conclusions:**

The associations identified between mPAP and mechanical ventilation variables in this analysis would suggest that classical ARDS lung protective strategies, including low tidal volume ventilation and permissive hypercapnia, may negatively impact the management of the subset of ARDS patients with associated right ventricular dysfunction or ACP. Additionally, respiratory rates above 17 cycles per minute show an incremental increase in mPAP. Therefore, increases in tidal volume (within the limitation of driving pressure < 18 cmH20) may represent a more right ventricular protective way to control CO2 and pH.

**Supplementary Information:**

The online version contains supplementary material available at 10.1186/s13054-022-04282-9.

## Background

The correlation between pulmonary hypertension and acute respiratory distress syndrome (ARDS) was described more than 45 years ago [1] and is associated with increased mortality [2–4]. High pulmonary artery pressures lead to an increase in right ventricular afterload, which is the main precipitant of acute cor pulmonale (ACP) [5]. ACP is diagnosed by echocardiogram and is generally defined by a right ventricle dilatation with septal dyskinesia [2]. Patients with ACP and ARDS have a progressively worse outcome as the severity of ACP increases [2–4]. This is observed even when lung protective ventilation is applied [3]. ACP may have an incidence of up to 28% in patients with ARDS [3, 5]. The previously proposed ACP risk score [3] was developed to aid the diagnosis of ACP in the context of ARDS. Identifying other risk factors for the development of ACP may improve diagnosis and institution of specific treatments, such as ventilator adjustments [6] and proning [7].

The relationship between indices of mechanical ventilation and pulmonary artery pressures remains ill-defined in ARDS. Specifically, as our understanding of mechanical ventilation has progressed, there is a greater understanding of the impact of high driving pressures and mechanical power [8, 9] in perpetuating lung injury. How these two parameters interact with pulmonary artery pressure remains poorly studied. This is, in part, related to the pulmonary artery catheter (PAC) falling out of favour in the majority of general intensive care units in the 2000s following the publication of several papers looking at their impact on patient outcomes [10, 11].

Consequently, we performed a post hoc analysis of the Fluid and Catheters Treatment Trial (FACTT) trial [11, 12] to investigate the associations between mechanical ventilation indices in ARDS patients and the development of pulmonary hypertension. This randomised study was a two-by-two factorial design trial comparing ARDS patients receiving liberal or conservative fluid management strategies and treatment guided by PAC’s or central venous catheter (CVC) protocols. This study, published in 2006, assigned 517 patients to receive PAC protocolised fluid administration and 480 patients to receive CVC protocolised fluid administration. The trial concluded that PAC-guided therapy did not improve survival or organ function but was associated with more complications than CVC-guided therapy.

## Methods

We performed a post hoc analysis of the FACTT database to identify ARDS patients who had a PAC inserted and pulmonary artery pressure readings recorded. We excluded any patient with a PAC inserted who was spontaneously breathing, as driving pressure and mechanical power is not validated in this cohort. Spontaneously breathing patients were identified as those who had a set respiratory rate lower than the total respiratory rate recorded.

After exclusions we identified 359 patients of the 501 patients from the original trial who had a PAC inserted, were not spontaneously breathing and had data on both pulmonary artery pressure and mechanical ventilation. In total, 120 patients were spontaneously breathing (set and measured rate differed) at all data points and were excluded. Thirty-two patients were excluded as they had no PAC catheter readings or missing respiratory rate data did not allow determination of spontaneous/mandatory breathing.

We calculated mean pulmonary artery pressure (mPAP) from the database by the following calculation;

$$\begin{aligned} {\text{mPAP}} & \, = \, 1/3\,{\text{systolic}}\,{\text{pulmonary}}\,{\text{artery}}\,{\text{pressure}}\,\left( {{\text{sPAP}}} \right) \\ & \quad + \, 2/3\,{\text{diastolic}}\,{\text{pulmonary}}\,{\text{artery}}\,{\text{pressure}}\,\left( {{\text{dPAP}}} \right). \\ \end{aligned}$$

A mPAP measurement of > 20 mmHg is considered to be diagnostic of pulmonary artery hypertension according to recently published international consensus guidelines [13].

The physiological and mechanical ventilation variables analysed to look for an association with mPAP were tidal volume, respiratory rate, total positive end expiratory pressure (PEEP), peak pressure, mean airway pressure, P/F ratio, arterial blood pH level and arterial PaCO_2_ level. We also compared three derived variables of ventilation in the analysis, these were:

1. Driving pressure. Calculated by Driving pressure = (Pplat − PEEP)
2. Mechanical power of ventilation. Calculated by Mechanical power (MP) = (MP = 0.098 × respiratory rate × tidal volume in litres × (Peak pressure − Driving pressure/2)
3. Mechanical power indexed to compliance. Calculated by Mechanical Power/Compliance (Compliance = Tidal volume/Driving Pressure)

Power was indexed to compliance as this has previously been demonstrated to have a stronger association with mortality than MP alone [14].

### Statistical analysis

We performed three consecutive statistical analyses; a univariate analysis, a multivariate regression analysis to assess for independent associations and an analysis to assess for nonlinear associations. Finally, we performed a sensitivity analysis to assess the impact of missing data.

Analysis was conducted using Stata version 17 (StataCorp, Texas). Variables were log-transformed where appropriate to meet the model assumptions. Associations between mPAP and mechanical ventilation parameters were assessed using Pearson correlation coefficients. As some patients had repeated measurements, we also used a structural equation modelling approach based on standardised data which allowed us to obtain robust standard errors for the correlation and *p* values which accounted for clustering. Both approaches give identical correlation coefficients.

To assess whether the observed associations were independent, we performed variable selection using an all-subsets approach to identify the best multiple regression model according to Akaike’s information criteria.

To check for nonlinearity, we selected the best-fitting model according to the Bayesian information criterion (BIC) from linear, quadratic, fractional polynomial and restricted cubic spline models (see Additional file 1: Appendix B). Improvement above the linear model was tested using likelihood ratio tests. Where effects were nonlinear, we calculated the inflexion point of the curve. Changes in mPAP were calculated for a 1% or one unit change in mechanical power and respiratory rate by fitting a linear regression model for values above the inflexion point. This model used a robust standard error with clustering on patient ID. A significance level of 0.05 was used.

Complete case analysis was used. In addition, we conducted a sensitivity analysis to assess the impact of missing data. Missing values were imputed by multiple imputation using multivariate normal regression and a Markov chain Monte Carlo method (see Additional file 1: Appendix A—Tables S4 and S5). Ten imputed datasets were created and parameter estimates combined after accounting for the variability between imputations.

## Results

A total of 359 patients were identified with between 1 and 5 observations each (Table 1). The sample includes *N* = 855 observations with pulmonary artery pressure recordings and mechanical ventilation data (after excluding 2511 observations where patients were breathing spontaneously).

**Table 1:** Ventilation and mPAP observations

Variable	Mean (SD)	Median [IQR]	Number of observations	Number of patients
mPAP (mmHg)	29.4 (7.6)	28.7 [24–34]	840	359
P/F ratio (mmHG)	161.2 (81.0)	141.5 [135–147.3]	767	334
Blood pH level	7.37 (0.09)	7.38 [7.31–7.43]	769	334
PaCO2 level (mmHg)	46.5 (12.8)	45 [37.3–52]	768	334
Tidal volume (mls)	394.8 (85.2)	400 [330–450]	819	341
PEEP (cmH20)	9.8 (4.0)	10 [8–12]	840	350
Respiratory rate (cpm)	27.7 (6.5)	28 [23–35]	835	346
Plateau pressure (cmH20)	26.2 (7.5)	25 [21–30]	778	334
Peak airway pressure (cmH20)	33.7 (8.9)	33 [28–39]	824	343
Mean airway pressure (cmH20)	16.4 (5.5)	16 [13–19]	778	332
Driving pressure (cmH20)	16.3 (6.4)	15 [13–19]	777	334
Mechanical power (joules/min)	27.7 (12.4)	26.0 [19.1–34.0]	762	328
Mechanical power indexed to compliance (joules/min/ml/cmH2O)	1.24 (0.93)	1.03 [0.23–1.54]	762	328

Of the 359 patients identified, 331 patients had at least one mPAP value greater than 20 mmHg, suggesting a high prevalence of pulmonary artery hypertension.

### Univariate analysis

All unadjusted mechanical ventilation variables were significantly correlated with mPAP. Mean PAP decreased with increasing P/F ratio, pH level and tidal volume, whilst it increased for the other ventilation variables (Table 2).

**Table 2:** Correlation of mPAP with mechanical ventilation variables

Variable	Unadjusted		Adjusted for clustering		*N*
	Correlation	*p* value	Correlation (robust SE)	*p* value**	
P/F ratio*	− 0.37	< 0.001	− 0.37 (0.04)	< 0.001	754
Blood pH level	− 0.33	< 0.001	− 0.33 (0.04)	< 0.001	756
PaCO2 level*	0.24	< 0.001	0.24 (0.04)	< 0.001	755
Tidal volume	− 0.10	0.004	− 0.10 (0.04)	0.02	805
PEEP*	0.36	< 0.001	0.36 (0.04)	< 0.001	826
Respiratory rate	0.28	< 0.001	0.28 (0.04)	< 0.001	821
Plateau pressure*	0.35	< 0.001	0.34 (0.04)	< 0.001	765
Peak airway pressure	0.29	< 0.001	0.29 (0.04)	< 0.001	810
Mean airway pressure*	0.38	< 0.001	0.38 (0.04)	< 0.001	766
Driving pressure	0.18	< 0.001	0.18 (0.05)	< 0.001	764
Mechanical power*	0.25	< 0.001	0.25 (0.04)	< 0.001	749
Mechanical power indexed to compliance	0.31	< 0.001	0.1 (0.04)	< 0.001	749

^*^Log-transformed variable

^**^Adjusted for clustering of results within patients

For the driving pressure variable, we obtained correlations both before and after excluding outliers (DP > 37, *n* = 10) and obtained similar results (*r* = 0.18 before and *r* = 0.19 after exclusions).

**Fig. 1:**
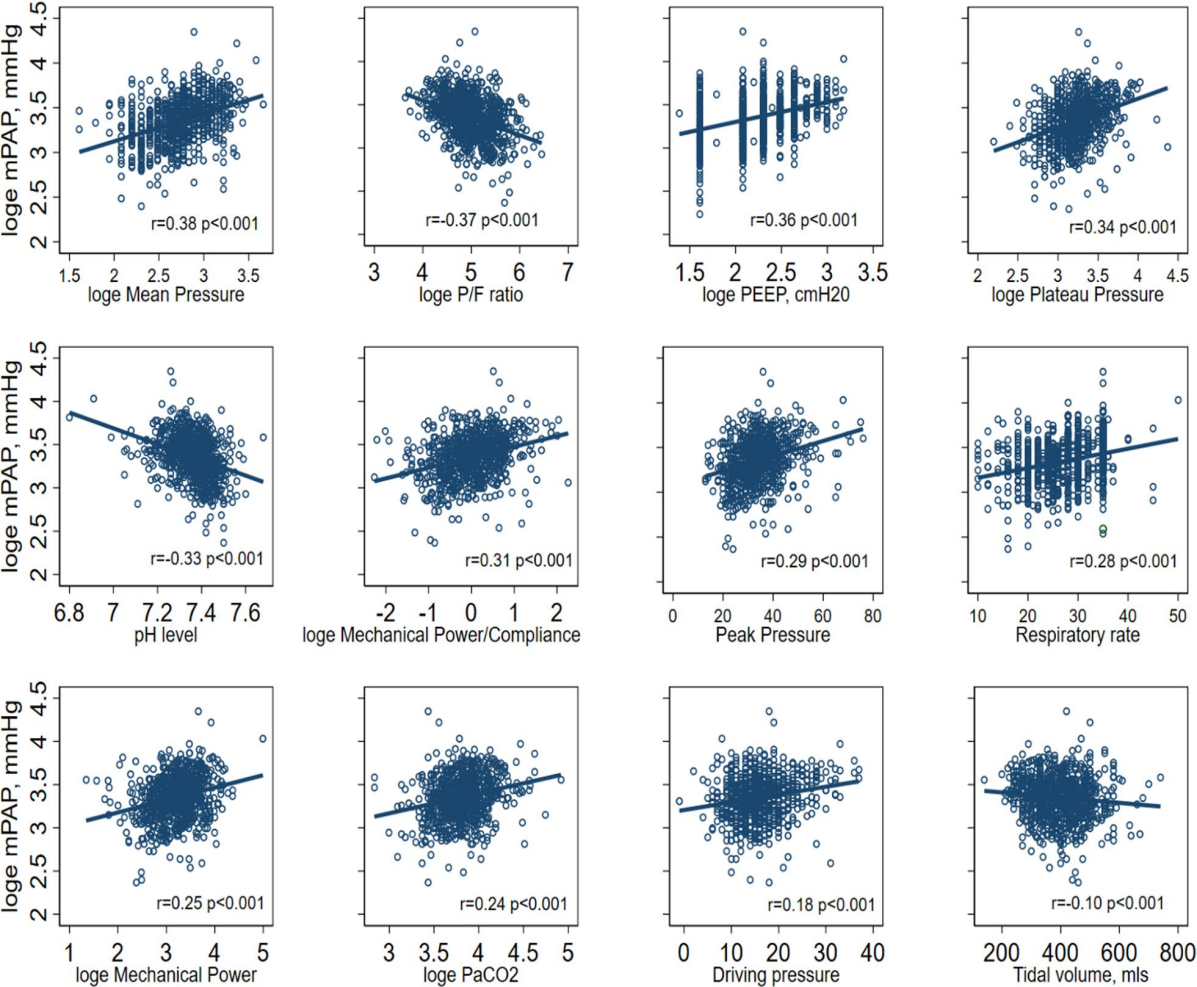
Correlations between mPAP and mechanical ventilation variables

The correlation of mPAP with each mechanical ventilation variable was plotted and ordered in decreasing levels of correlation with mean airway pressure having the highest correlation and tidal volume having the lowest (Fig. 1).

As it was counter-intuitive that increasing tidal volume would decrease mPAP, we analysed the associations with tidal volume further. As tidal volume increased, the respiratory rate decreased, and this association with reduced respiratory rate had a correlation = − 0.29. When adjusted for this correlation, there is no significant association between mPAP and tidal volume (*r* = − 0.02, *p* = 0.57).

### Multivariate analysis

Variable selection was used to identify variables independently associated with mPAP. Blood pH level, P/F ratio, PACO_2_ level, mean airway pressure and mechanical power indexed to compliance were identified as independent predictors of mPAP (Table 3).

**Table 3:** Standardised effect sizes from best-fitting multiple regression model for mPAP

	B (se)	*p* value
Blood pH level	− 0.17 (0.04)	< 0.001
P/F ratio*	− 0.17 (0.04)	< 0.001
PaCO2 level*	0.08 (0.04)	0.02
Mean airway pressure*	0.12 (0.05)	0.02
Mechanical Power indexed to compliance	0.14 (0.04)	0.001

^*^Log-transformed data

### Testing for nonlinearity

Associations did not differ from linearity except for 4 variables for which the fractional polynomial was the best-fitting model. These were mechanical power (*p* = 0.01 compared to the linear model), respiratory rate (*p* = 0.04), peak pressure (*p* = 0.03) and mean airway pressure (*p* = 0.01). (For further information on nonlinear correlations, see Additional file 1: Appendix B—Table S6.)

**Fig. 2:**
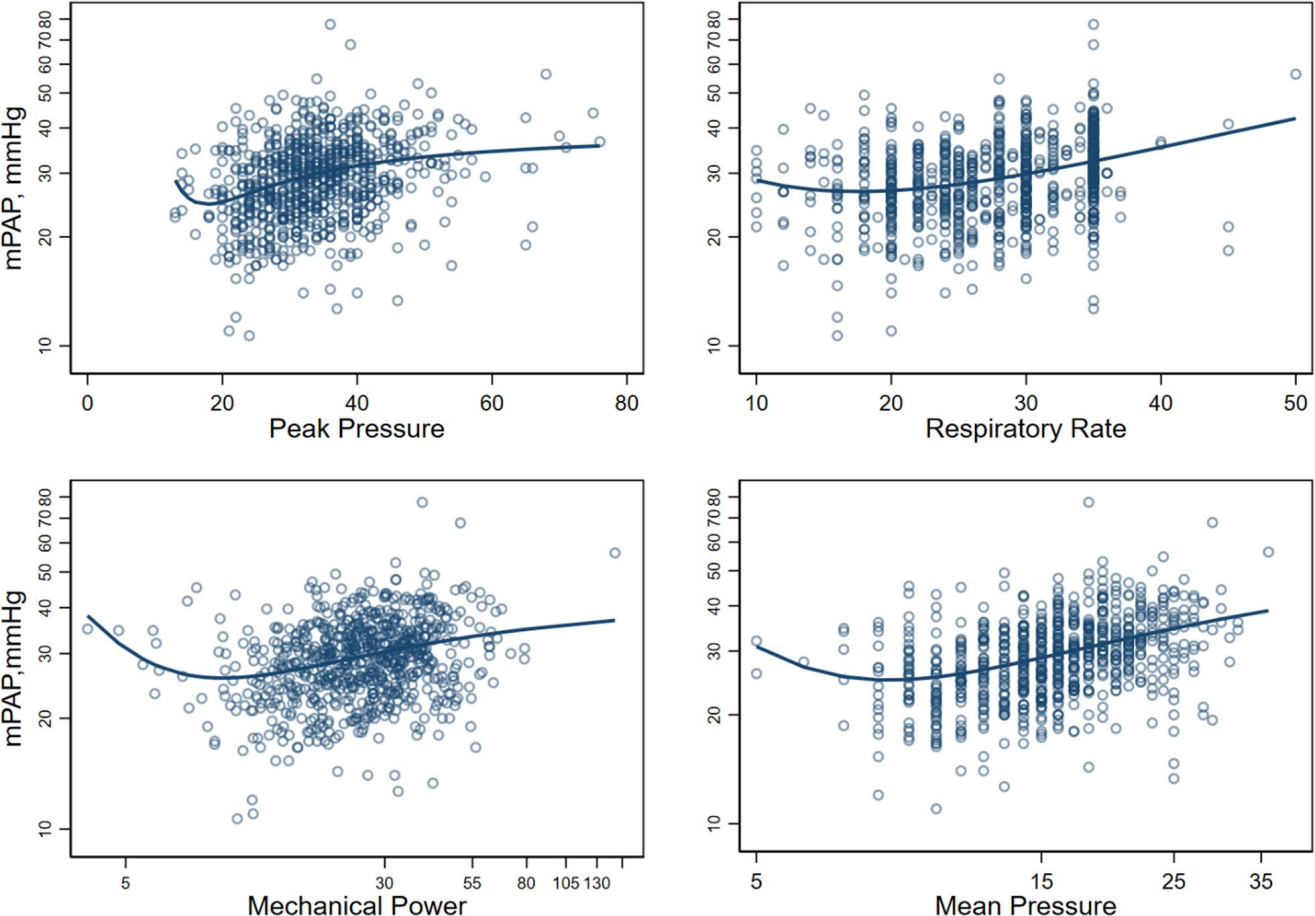
Nonlinear correlations between mPAP and mechanical ventilation variables

We have changed the x-axis scale for the log-transformed data for mechanical power and mean pressure to show the original units (Fig. 2). The inflexion points for each variable are:-

- Peak pressure = 17.7 cmH20
- Respiratory rate = 16.8 cycles per minute
- Mechanical power = 8.8 J/min
- Mean airway pressure = 10.0 cmH20

### Extended nonlinear analysis

Two nonlinear variables associated with mPAP were assessed in more detail. Mechanical power, the energy delivered by the ventilator, is a relatively recent concept that incorporates many ventilator variables.

**Fig. 3:**
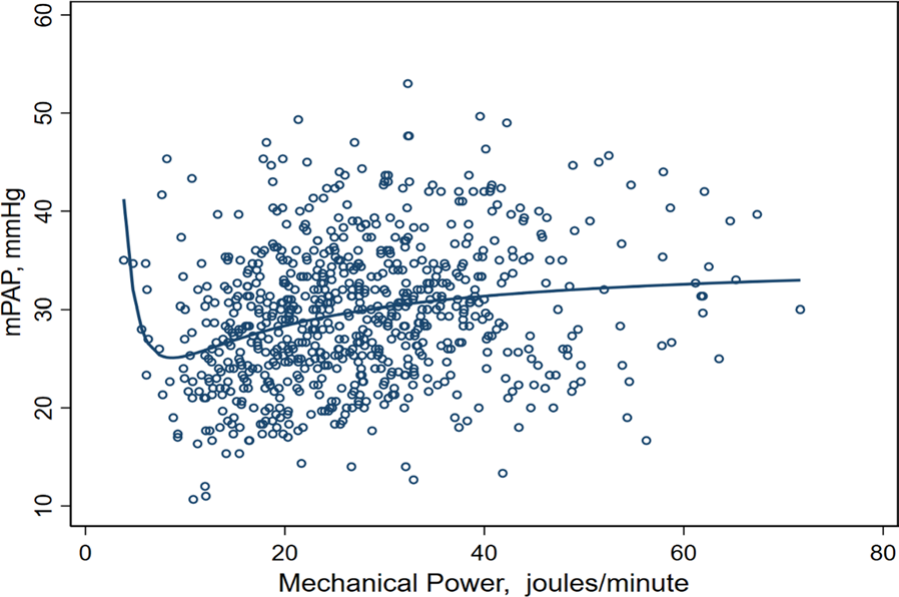
Correlation between mPAP and mechanical power

A 1% change in mechanical power results in a 0.18% change in mPAP (95% CI 0.12–0.23) *p* < 0.001, or an increase in mPAP of 0.17 mmHg, if we model the linear effect above the inflexion point of 8.8 J/min (Fig. 3).

**Fig. 4:**
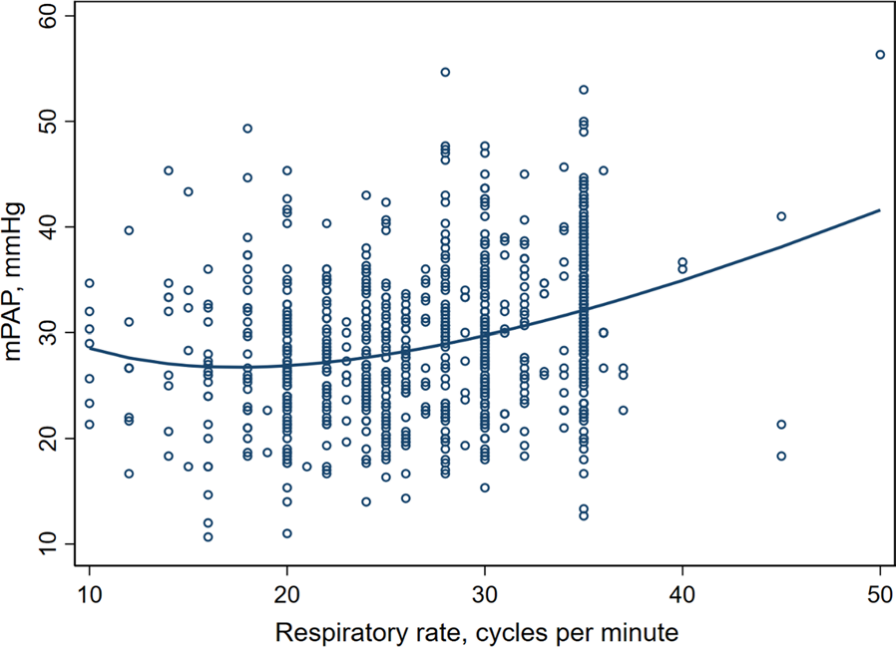
Correlation between mPAP and respiratory rate

Respiratory rate is frequently increased at the bedside in patients where ARDSNet ventilation is being applied. A 1 unit increase in respiratory rate results in a 1.2% change in mPAP (95% CI 0.9–1.6%) *p* < 0.001, or an increase in mPAP of 0.37 mmHg, if we model the linear effect above the inflexion point of 16.8 CPM (Fig. 4).

## Discussion

In this post hoc analysis of the FACCT trial, we observed that 331 of 359 or 92% of patients had an mPAP exceeding 20 mmHg (3.3 kPa). This incidence is similar to a prospective study by Beiderlinden et al. [15] of 103 patients who reported a pulmonary hypertension incidence of 92.2% in ARDS patients. As ARDS-related pulmonary hypertension progresses, the frequency of ARDS-related Acute Cor Pulmonale (ACP) increases, occurring in up 28% of all ARDS patients [16]. Importantly, it is associated with increased mortality [16].

As the echocardiographic features of ACP are reversible and may be treated by a range of therapeutic modalities, including adjustment of ventilator settings, prone positioning, fluid management and vasoactive drugs early diagnosis is important. Dessap et al. performed a prospective study of 752 patients with moderate-to-severe ARDS receiving lung protective ventilation and found that 22% had evidence of ACP [3]. Splitting patients into a derivation and validation cohort, they identified four variables: pneumonia as a cause of ARDS, driving pressure ≥ 18 cm H_2_O, arterial oxygen partial pressure to fractional inspired oxygen (PaO_2_/FiO_2_) ratio < 150 mmHg and arterial carbon dioxide partial pressure ≥ 48 mmHg, which comprised the ACP risk score. The ACP risk score was subsequently validated by Li et al. [17] in a retrospective analysis of 2434 patients. Additionally, the ACP score correlated with an increased incidence of pulmonary hypertension in patients with ARDS, with each increase in ACP score associated with an increase in the prevalence of pulmonary hypertension.

We have used mPAP as a marker of pulmonary hypertension [13] and as mPAP reflects right ventricular afterload, which is a major determinant of ACP, a surrogate marker of the risk of ACP. We investigated the association of mPAP with several ventilation parameters in ARDS from our previously described derivation cohort. All the ventilation parameters demonstrated a significant correlation with mPAP in our initial univariate analysis, except tidal volume (once adjusted for respiratory rate).

The subsequent multivariate analysis demonstrated that the blood pH level, P/F ratio, PaCO_2_ level, mean airway pressure and the mechanical power indexed to compliance were independently associated with mPAP. All of these variables represent potential independent surrogate markers of the risk of ACP. Amongst these variables, the mechanical power indexed to compliance, which is a representation of the energy applied per minute to the amount of well-aerated lung tissue in ARDS patients, has never previously been shown to have an association with mPAP. This may help to partially explain the results of a previous study in 2020 by Copolla et al. [18] demonstrating that mechanical power normalised to compliance is independently associated with intensive care mortality in ARDS patients.

To date, the only published description of right ventricular protective mechanical ventilation was by Paternot et al. in 2016 [6]. In this opinion article, they describe an approach targeting four variables; plateau pressure < 28 cmH_2_0, driving pressure < 18 cmH_2_0, P/F ratio > 150 mmHg and PaCO_2_ < 48 mmHg. The optimal PEEP for this approach was stated to be controversial but was described as the PEEP associated with the best PaO_2_/FIO_2_ ratio without altering compliance. In our *post* hoc analysis, all the components suggested by Paternot et al. have demonstrated a strong correlation in our univariate analysis with mPAP. Additionally, the P/F ratio and PaCO_2_ were demonstrated to be independently associated with the prevalence of raised mPAP. Both of these results add further validation to this approach.

Given the data from this analysis, it is possible to further elucidate what represents right ventricular protective ventilation. Specifically, the addition of respiratory rate appears an important variable for consideration in any future approach. Respiratory rates above 17 cycles per minute demonstrated an incremental increase in mPAP in the final extended nonlinear analysis. The respiratory rate is also an integral part of the mechanical power equation, which when indexed to compliance is independently associated with mPAP. Therefore, increases in tidal volume (within the limitation of driving pressure < 18 cmH20) may represent a more right ventricular protective way to control CO_2_ and pH. This is consistent with a prospective study by Vieillard-Baron et al. [19] which concluded that high respiratory rates in ARDS patients did not improve CO_2_ clearance, produced dynamic hyperinflation and impaired right ventricular ejection.

There are a number of weaknesses to this study. The retrospective nature of this post hoc analysis means these data are hypothesis generating only. We used mean pulmonary artery pressure as a marker of pulmonary hypertension in ARDS, to align with international definitions [13]. However, we appreciate there are other markers of PVR that could have been used in its place [20]. As the other arm of the trial used for this post hoc analysis was fluid administration in ARDS, there is a risk of unequal fluid resuscitation in our derivation cohort which could also affect our findings [11]. Another weakness of this analysis is that the cohort identified had a very high mortality rate and we were unable to perform a mortality analysis with enough statistical power due to this imbalance.

It should also be noted that the pulmonary haemodynamic data used for this analysis have come from this singular trial, and that data from PACs are subject to errors both in measurement and interpretation of their waveform and generated haemodynamic data as demonstrated by Parviainen et al. [21]. This trial did not undertake routine echocardiography on all participants to be able to explore whether there would be echocardiographic surrogate findings along the same lines as the PAC data. This is particularly pertinent due to the prominence and ubiquity of echocardiography in current critical care practice, as well as having dedicated echocardiographic protocols and definitions of right heart dysfunction and ACP. Whether the data used to inform such a decision-making process in the future should be echocardiogram finding-driven or PAC-driven is beyond the scope of a *post hoc* analysis. However, a recent publication by Cloverdale et al. [22] has questioned whether there could be a role for the return of the PAC to intensive care practice.

In conclusion, the associations identified from this analysis would suggest that classical ARDS lung protective strategies, including low tidal volume ventilation and permissive hypercapnia, may negatively impact the management of the subset of ARDS patients with associated right ventricular dysfunction or ACP. The index of suspicion of such dysfunction, coupled with the ACP score, may help generate a dedicated study to evaluate for this pathology, and subsequently tailor ventilatory management not just to a lung protective strategy, but to a lung and right heart protective one. The previously described right ventricular protective ventilation strategy by Paternot et al. has been validated. The addition of respiratory rate to future right ventricular protective strategies, given its strong association with mPAP, its integral part in the mechanical power equation and its inflexion point at 17 cycles per minute, is strongly recommended.

## Supplementary Information

**Additional file 1.** Supplementary Appendix A & B.

## Data Availability

This manuscript was prepared using FACTT research materials obtained from the NHLBI Biologic and Data Repository Information Coordinating Centre and does not necessarily reflect the opinions or views of the FACTT or NHLBI.
