# Supplementary material for: Mechanical ventilation variables associated with high pulmonary artery pressures in ARDS patients: a post hoc analysis
Source: Crit Care. 2022 Dec 21;26:396. doi: 10.1186/s13054-022-04282-9 (PMC9773567; doi:10.1186/s13054-022-04282-9)
Supplement: Supplementary file 1 — Additional file 1. Supplementary Appendix A & B. [file 13054_2022_4282_MOESM1_ESM.docx]

**ADDITIONAL FILE 1**

**APPENDIX A**

**Table S4:- Missing data**

There is missing data for each variable between 1.8% and 11%.

|  | Number missing (%) |
| --- | --- |
| MPAP* | 15 (1.8%) |
| P/F ratio* | 88 (10.3%) |
| Blood pH level | 86 (10.1%) |
| PaCO2 level* | 87 (10.2%) |
| Tidal volume | 36 (4.2%) |
| PEEP* | 15 (1.8%) |
| Respiratory rate | 20 (2.3%) |
| Plateau pressure* | 77 (9.0%) |
| Peak pressure | 31 (3.6%) |
| Mean pressure* | 78 (9.1%) |
| Driving pressure | 78 (9.1%) |
| Mechanical Power* | 93 (10.9%) |
| Mechanical Power indexed to compliance | 94 (11.0%) |

*log-transformed data

**Table S5:- Sensitivity analysis: correlations after multiple imputation**

|  | **Unadjusted** | |
| --- | --- | --- |
| **Variable** | **Correlation (SE)** | **P value** |
| P/F ratio* | -0.37 (0.04) | <0.001 |
| Blood pH level | -0.34 (0.04) | <0.001 |
| PaCO2 level* | 0.23 (0.04) | <0.001 |
| Tidal volume | -0.10 (0.04) | 0.02 |
| PEEP* | 0.35 (0.04) | <0.001 |
| Respiratory rate | 0.27 (0.04) | <0.001 |
| Plateau pressure* | 0.35 (0.04) | <0.001 |
| Peak pressure | 0.29 (0.04) | <0.001 |
| Mean pressure* | 0.38 (0.04) | <0.001 |
| Driving pressure | 0.18 (0.04) | <0.001 |
| Mechanical Power* | 0.26 (0.05) | <0.001 |
| Mechanical Power indexed to compliance | 0.31 (0.04) | <0.001 |

*log-transformed data

Missing values were imputed by multiple imputation using multivariate normal regression and a Markov chain Monte Carlo method. 10 imputed datasets were created and parameter estimates combined after accounting for the variability between imputations.

Rubin, D. B. 1987. Multiple Imputation for Nonresponse in Surveys. New York: Wiley.

**APPENDIX B**

Correlations

To compare linear and non-linear correlations we used the R-squared, which is the proportion of variance in mPAP that is explained by each non-linear variable when looking at non-linear effects **(Table 5).** For the linear model R-squared is the correlation squared.  We have demonstrated the R2 for the linear and non-linear models in the table below.

**Table S6:- Linear and non-linear correlations**

| **Variable** | **R2**  **linear** | **R2 non-linear** |
| --- | --- | --- |
| Tidal volume | 1.0% |  |
| PEEP* | 12.8% |  |
| P/F ratio* | 13.6% |  |
| Blood pH level | 11.2% |  |
| PaCO2 level* | 5.6% |  |
| Driving pressure | 3.3% |  |
| Mechanical Power* | 6.4% | 7.8% |
| Mechanical Power indexed to compliance | 9.6% |  |
| Respiratory rate | 7.7% | 8.6% |
| Plateau pressure* | 12.1% |  |
| Peak pressure | 8.5% | 9.5% |
| Mean pressure* | 14.7% | 15.9% |

*log-transformed data
